# Correction Without Consciousness in Complex Tasks: Evidence from Typing

**DOI:** 10.5334/joc.202

**Published:** 2022-01-07

**Authors:** Svetlana Pinet, Nazbanou Nozari

**Affiliations:** 1 Department of Neurology, Johns Hopkins University, Baltimore, US; 2 BCBL. Basque Center on Cognition, Brain and Language, Donostia-San Sebastian, ES; 3 Department of Psychology, Carnegie Mellon University, Pittsburgh, US; 4 Center for Neural Basis of Cognition (CNBC), US

**Keywords:** errors, repair, correction, typing, awareness

## Abstract

It has been demonstrated that with practice, complex tasks can become independent of conscious control, but even in those cases, repairing errors is thought to remain dependent on conscious control. This paper reports two studies probing conscious awareness over repairs in nearly 15,000 typing errors collected from 145 participants in a single-word typing-to-dictation task. We provide evidence for subconscious repairs by ruling out alternative accounts, and report two sets of analyses showing that a) such repairs are not confined to a specific stage of processing and b) that they are sensitive to the final outcome of repair. A third set of analyses provides a detailed comparison of the timeline of trials with conscious and subconscious repairs, revealing that the difference is confined to the repair process itself. We propose an account of repair processing that accommodates these empirical findings.

## Introduction

Monitoring performance for prevention, detection, and correction of errors is an integral part of most behaviors. It has been argued that highly practiced tasks rely less on conscious processes that control and regulate them (Anderson, 1982; G. D. Logan, 1988). But can monitoring and repair processes themselves become independent of conscious awareness? There is evidence that error detection can be carried out subconsciously; for example, error related negativity (ERN), an EEG marker of error detection, is also found in subconsciously reported errors, although its magnitude may be modulated by consciousness (Nieuwenhuis, Ridderinkhof, Blom, Band, & Kok, 2001; Wessel, 2012). Less is known about the dependence of error correction on consciousness. Some evidence suggests that corrections can be made without any conscious recollection of having committed an error or having attempted a repair. This evidence, however, comes from simple tasks that are themselves not subject to consciousness, e.g., saccadic eye movements (Nieuwenhuis et al., 2001). It is thus an open question whether repairing errors in more complex tasks can be carried out without conscious awareness. We answer this question by examining repairs in typing.

### Why typing?

Typing is an intriguing task combining higher-level semantico-lexical processes with lower-level motor processes (G. D. Logan & Crump, 2011; Pinet & Nozari, 2018; Pinet, Ziegler, & Alario, 2016; Yamaguchi, Crump, & G. D. Logan, 2013). On the one hand, the generative nature of language production limits repetitive behavior in typing. On the other hand, the behavior can be highly practiced to the degree that implicit typing performance in expert typists is far better than the explicit knowledge over the task details such as the absolute and relative positions of the keys (Liu, Crump, & G. D. Logan, 2010; Snyder, Ashitaka, Shimada, Ulrich, & G. D. Logan, 2014). This combination makes typing a hallmark of a complex goal-oriented task with an automatized component (see G. D. Logan, 2018, for an implementation of such a “controlled automatic” process through a context retrieval and updating mechanism).

At the same time, compared to a similarly complex task of spoken production (<1% errors), typing is more error-prone (~10–15%, Pinet & Nozari, 2018). This provides many opportunities for observing repairs. The repair process itself is also unique in several ways: a) it is discrete in that it requires replacing a whole segment (i.e., a letter) with a new one. b) It requires erasing the mistake by the use of a unique operation (pressing the backspace) before applying the repair. c) Repairs can be implemented immediately after the error segment. This requires not only stopping the current activity but its resumption from the right point after repair, which makes tracking the point of repair critical. In short, apart from typing itself being a complex generative task, repairing typing errors is itself a complex process, requiring stopping, deleting, inserting a new segment, and resuming production from a specific point. Due to such complexities, even models such as G. D. Logan (2018), which proposes an automatic component for typing, maintain that repairs must require conscious deliberation and application of top-down control. This paper revisits this position.

### What is at stake?

Whether monitoring in a complex task such as language production does or does not have a subconscious component leads to very different theoretical accounts. On the error detection side, for example, there have been significant theoretical advances over the past decade that have offered subconscious alternatives (Guenther, 2016; Hickok, 2012; Nozari, Dell, & Schwartz, 2011; see Nozari, 2020 for a review) to the classic comprehension-based monitor (Levelt, 1983). In contrast, a clear mechanism for repairs in language production is yet to be established (Gauvin & Hartsuiker, 2020). An exception is the extension of Guenther’s forward model, which corrects for the deviation between motor plans and perceptual targets through an inverse model (Guenther, 2016; Wolpert & Kawato, 1998). As useful as such a mechanism is for adjusting the low-level aspects of production, it is unlikely to underlie the repair of larger segments such as phonemes, letters, and words (see Nozari, 2020 for detailed arguments). This, in turn, calls for a repair mechanism applicable to higher levels of the production system, i.e., repairs of segments (e.g., letters) and words. Two general mechanisms have been proposed in the literature: a) re-instating the plan. G. D. Logan (2018) proposes that a typing repair requires re-instating the initial word command before the automatic component can take over (G. D. Logan, 2018, p. 475). Since such a process requires conscious planning, it naturally puts repairs outside of the domain of subconscious processing. b) Local switches. An alternative is a process that does not require re-instating the plan, but instead makes local switches in the automatic part of the production process. An example is replacing a segment or a word with the next most highly activated representation upon the generation of an error signal (e.g., Nooteboom & Quené, 2020; Nozari, Martin, & McCloskey, 2019). This process could operate without conscious awareness.

Currently, the empirical evidence for the degree to which conscious control is involved in repairs in language production is mixed. On the one hand, there is indirect evidence pointing to a potential automatic component for repairs: corrections can be very quick and efficient (Nooteboom & Quené, 2019, 2020), and studies in some populations like young children and individuals with aphasia suggest that such corrections may be carried out without explicit awareness over the error or the repair process (e.g., Clark, 1978). On the other hand, some findings have been taken as evidence that repairs are subject to attentional control. For example, when participants become more error prone, they tend to correct a higher proportion of their errors (Levelt, 1983; Nozari et al., 2019). But being subject to controlled processing does not rule out an underlying automatic process. In a seminal paper, Moors and de Houwer (2006) reviewed the various approaches to defining automaticity (e.g., feature-based vs. construct-based) and discussed the disagreement even among the proponents of the feature-based approach on whether all or a subset of critical features should be present for a process to be considered automatic, giving rise to the all-or-none vs. decompositional views of automaticity, respectively. Following the logic of the decompositional views, it is perfectly plausible for a complex operation to only partially meet the criteria for automaticity. While there is some disagreement about what the key criteria are, subconscious processing frequently makes the list; if a task can be performed without conscious awareness, it falls closer to automaticity on the spectrum than tasks that require conscious processing for completion.

While there are some implicit ways of probing consciousness over errors, such as using EEG components that have been empirically linked to conscious judgments (e.g., late positivity; Pinet & Nozari, 2020), the most well-established method has been the use of metacognitive judgements. This can take several forms: pressing a button only in the case of an error (Nieuwenhuis et al., 2001), classifying trials as error or correct (Charles et al., 2013), or specifying the type of error (e.g., which distractor was chosen, Di Gregorio et al., 2016). The assumption here is that although one can have an implicit sense of how one is performing in a given task, a deliberate metacognitive judgment about performance requires consciousness. If this cannot be achieved, the information from the primary task is deemed inaccessible to consciousness, at least to the level required for forming the basis of a clear conscious judgment.

### The current study

This paper reports two studies probing conscious awareness over repairs in about 15,000 typing errors from a single-word typing-to-dictation task. It is important to note that the question is not whether most repairs are subconscious, but rather if any repairs are subconscious. In other words, unlike most studies where the probability of an event is of great interest, what is critical here is the possibility of an event. The reason is that establishing the occurrence of subconscious corrections calls for mechanisms that are inherently different from current proposals of consciously restarting the production process and invites new theoretical accounts. The critical point, in turn, is to establish that subconscious corrections, if any, are a) replicable, and b) not the product of alternative mechanisms such as forgetting a conscious act. This is the approach we follow in this paper. Once established, we report three sets of analyses exploring key characteristics of subconscious repairs, and end by proposing an account of correction processes that accommodates the empirical findings.

## Methods

### Corpus composition

We analyzed a corpus of typing errors collected from two experiments (study 1 and study 2). The experiments are similar in their general structure; thus, they are described under one methodology, and their data are merged for the analyses. Study 2 provides a replication and addresses some concerns in study 1.

### Participants

Participants were recruited via Amazon Mechanical Turk. They were consented under a protocol approved by the Institutional Review Board of Johns Hopkins School of Medicine and were compensated for their participation. Participation eligibility was determined by a screening test administered before participants were accepted into the study. This test had two parts. In the first part, participants typed 15 words to dictation (one at a time), with a relaxed deadline (5000 ms). In the second part, they typed 15 words to dictation (one at a time), under a shorter deadline (2000 ms). The beginning and end of the typing period for each word was indicated by short beeps. Participants had to reach 80% accuracy on the first part, and finish typing at least 80% of trials under the time limit (with minimum 50% accuracy) on the second part to be accepted into the study. The screening phase continued until the pre-determined number of participants was reached for each study (60 for study 1, and increased to 85 for study 2 to increase the chance of finding unconscious correction attempts).

Study 1 included 60 participants (25 males, 35 females), aged on average 38.0 ±10.4 years old (range 21–63). All had completed high school and 27 had a bachelor’s degree or higher. All but two were English native speakers. The two who were not, had learned English at the age of five. The sample had a mean typing speed of 75.3 ± 19.0 words per minute (range 49–126), and an accuracy of 93.3 ± 5.8% (range 70–100). All participants reported using seven or more fingers for typing. Seven (11%) reported looking at their fingers when typing and 22 (37%) reported having formal typing training. Participants had been typing for 21.0 ± 8 years (range 6–40) and typed an average of 3.6 ± 2.6 hours a day (range 0.5–12).

Study 2 comprised 85 participants (53 males, 32 females), aged on average 35.3 ± 9.7 years old (range 21–65). All had completed high school and 39 had a bachelor’s degree or higher. All were English native speakers. The sample had a mean typing speed of 75.5 ± 15.0 words per minute (range 45–118), and an accuracy of 94.2 ± 5.5 % (range 76–100). Twenty-nine participants reported having formal typing training, and 21 reported looking at their fingers when typing. Ten participants reported using six or fewer fingers, which could be a source of concern about their typing expertise. However, over the whole sample, the number of fingers participants reported using was not correlated with their typing speed or accuracy. Participants had been typing for 20.5 ± 8 years (range 2–42) and typed an average of 3.7 ± 2.6 hours a day (range 0–10).

Eight participants participated in both studies. They were included in the analysis of each individual dataset. However, to analyze the whole corpus of errors, only their entry from dataset 1 was included, so that only unique subjects were present in the corpus. The final corpus comprised data from 136 participants.

### Materials

For both datasets, stimuli were 7 and 8-letter words. Stimuli for dataset 2 were a subset of stimuli used for dataset 1 (see Procedures below for numbers). Log-transformed word frequency was kept under 3, based on the SUBTLEX database (Brysbaert & New, 2009), to increase task difficulty. No plural words, compound words or words that had homophones were selected. Auditory stimuli were recorded by a native English speaker.

### Procedures

The experiment was programmed using the jsPsych library (de Leeuw, 2015), embedded in an HTML environment. The Python library psiTurk (Gureckis et al., 2016) was used to handle participants’ recruitment and compensation. Participants performed the experiment online using their own computer and keyboard.

Dataset 1 refers to data collected under study 1. It was acquired over two sessions and comprised 300 trials per participant (it was part of a larger experiment comprising 1200 trials). Dataset 2 (i.e., data collected under study 2) was acquired over one session and contained 600 trials per participant. In both cases, the trial structure was similar and as follows (see ***Figure 1***): an auditory stimulus was presented, followed immediately by a short beep that served as a cue to start typing the word. Participants then had 1800 ms to type before they heard a lower pitched beep that marked the deadline. We buffered an additional 500 ms after the second beep to preserve some of the responses typed after the deadline. Participants could see what they typed on the screen in real time. Once the time was up, a prompt asked them to judge whether they had made an error or not by pressing ‘Y’ for yes, ‘N’ for no, and ‘A’ in case they did not produce a response. Instructions stressed that if they had made an error and corrected it, it still counted as an error and the appropriate response would be ‘yes’. If they did not submit an answer by 5000 ms, a prompt reminded them to respond faster. Participants could take a break every 50 trials.

**Figure 1:**
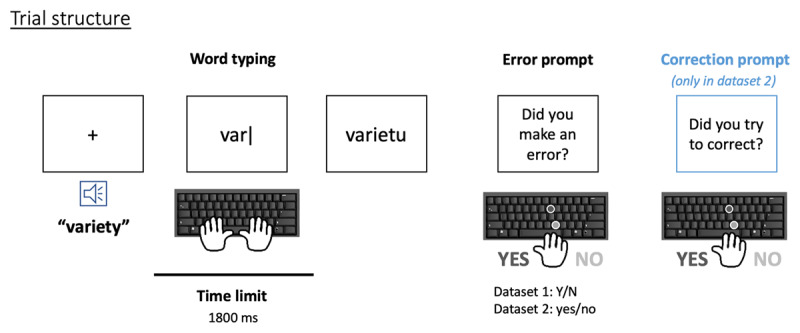
Trial structure for both datasets: a word typing period (under a time limit) is followed by an error prompt, and eventually a correction prompt (in Dataset 2 only).

Dataset 2 was collected with modified instructions to decrease, to the extent possible, the occurrence of unreported corrections by chance. Two changes were implemented: 1) The response format was changed from pressing a Y/N button to typing out the yes/no response. This eliminates the chance of a mistake due to pushing the wrong button. 2) A two-step procedure was implemented, in which participants first answered the question “Did you make an error when you first typed the word?”. If they answered ‘yes’, a second prompt asked them “Did you try to correct?”. To give them the best shot at reporting their errors, we went one step further this time: if their responses contained a backspace, even if they answered “no” to the first question, we asked them the second question. This strategy eliminated any chance that a misunderstanding about the nature of the question may have contributed to participants not reporting their errors.

### Data analyses

Any word that contained fewer keystrokes than the target word, and/or a keystroke not expected in the target word was considered an error. Reaction times (RTs) were calculated from the onset of the first beep (which followed immediately after the presentation of the auditory word) to the first keystroke. Inter-keystroke intervals (IKIs) were the time intervals between consecutive keystrokes and were averaged over each word. Hit Rates, Misses, Correct Rejections and False Alarms were calculated for each condition for each participant, and SDT parameters (d́’ and criterion) were estimated using the psycho R package (version 0.4.91, Makowski, 2018, R version 3.3.3). RTs and IKIs above or below 3SD of the mean of each participant’s RT and IKI distributions were removed for the analysis. Error detection rates were computed based on subjective reports (metacognitive prompt at the end of every trial). Since the use of a backspace is vital in any correction attempts, we have defined our measure of correction as such. Correction attempt rates were computed as the number of trials in which the backspace key was pressed at least once, divided by the total number of trials. Analyses were run using non-parametric statistical tests for categorical variables (Wilcoxon sign-rank tests) and linear mixed effect models for continuous variables (package lmerTests, version 3.0-1). Data and analysis scripts can be found here: *https://osf.io/jg3rm/*.

## Results

One participant (in study 2) never reported errors, meaning he had a discriminability index d’ of zero and a high response criterion (c = 2.82). Since error detection behavior is our variable of interest here, this participant was excluded from further analyses.

### Datasets description

We analyzed a corpus of 14,841 typing errors, 3,459 (20%) from dataset 1 and 11,382 (22.6%) from dataset 2. Error detection rates were 69% and 68%, and correction rates 29% and 37%, in datasets 1 and 2, respectively (***Figure 2***), with no significant differences between the two datasets. The mean IKI (i.e., time between two keystrokes) in dataset 1 was 166.1 ms/keystroke, ranging from 106.5 to 235.1 ms/keystroke. The average RT (i.e., time of the first keystroke) was 332.9ms, ranging from 198.7 to 543.5ms. In dataset 2, mean IKI was 161.9 ms/keystroke, ranging from 103.8 to 240.7 ms/keystroke. The average RT was 312.7ms, ranging from 213.1 to 453.5ms. Neither the mean IKIs, nor the RTs were significantly different in the two datasets. The RT analyses replicated the finding in both typing and spoken production (Crump & G. D. Logan, 2013) that errors (392.4 ± 103 ms) have significantly longer RTs than correct (318.1 ± 63 ms) words (*ß* = –55.9, *t* = –30.1, *p* < .001).

**Figure 2:**
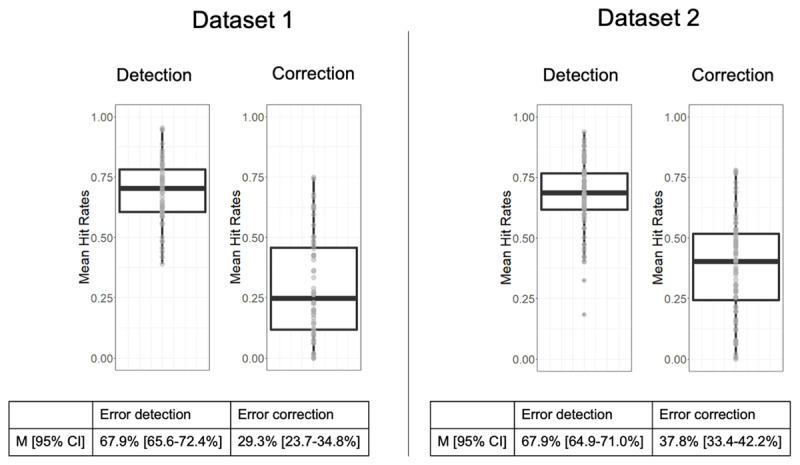
Distributions of hit rates for error detection and error correction attempt over subjects for each dataset. Confidence intervals (95%) are presented in square brackets.

### Unreported correction attempts

Unreported correction attempts were defined as trials that were not reported as containing an error, even though a backspace was used to change a typed letter. There were 173 (19.2% of 900 responses with a backspace) such trials in dataset 1 and 397 (10.3% of 3817 responses with a backspace) in dataset 2 (see ***Table 1***). ***Figure 3*** shows the variability in the proportions of unreported correction attempts between participants. They were evident in 63% of participants in dataset 1. Despite the measures taken to reduce the chance of missing a correction attempt, 82% of participants in dataset 2 still showed evidence of unreported correction attempts, meaning that switching Y/N to typed out responses did not eliminate unreported correction attempts. Separation of probing for error detection and correction attempt in dataset 2 also did not eliminate unreported correction attempts. Out of 3404 reported errors, 241 trials with backspace (7%) were still reported as not an attempt at correction. As a comparison, correction attempts were falsely reported on 825 trials without backspace (19%). Finally, clueing participants to the fact that an error has been made by asking them about correction attempts on all error trials with a backspace, whether reported and unreported, did not eliminate unreported correction attempts. Recall that in dataset 2, if participants responded “no” to the first question about error commission but had used the backspace, they were still presented with the second question about attempting to correct. Out of such 394 correction attempts, 241 (61%) remained unreported.

**Table 1:** Raw counts and percentages of trials with correction attempts, broken down by whether an error was reported or not. Cells in bold cells correspond to unreported correction attempts (percentage computed out of the total number of trials with correction attempt).


	DATASET 1		DATASET 2	

	ERROR REPORTED	ERROR NOT REPORTED	ERROR REPORTED	ERROR NOT REPORTED

Correction attempt	727 (80.8%)	**173 (19.2%)**	3423 (89.7%)	**394 (10.3%)**

No correction attempt	1647 (64.4%)	912 (35.6%)	3998 (56.1%)	3128 (43.9%)


**Figure 3:**
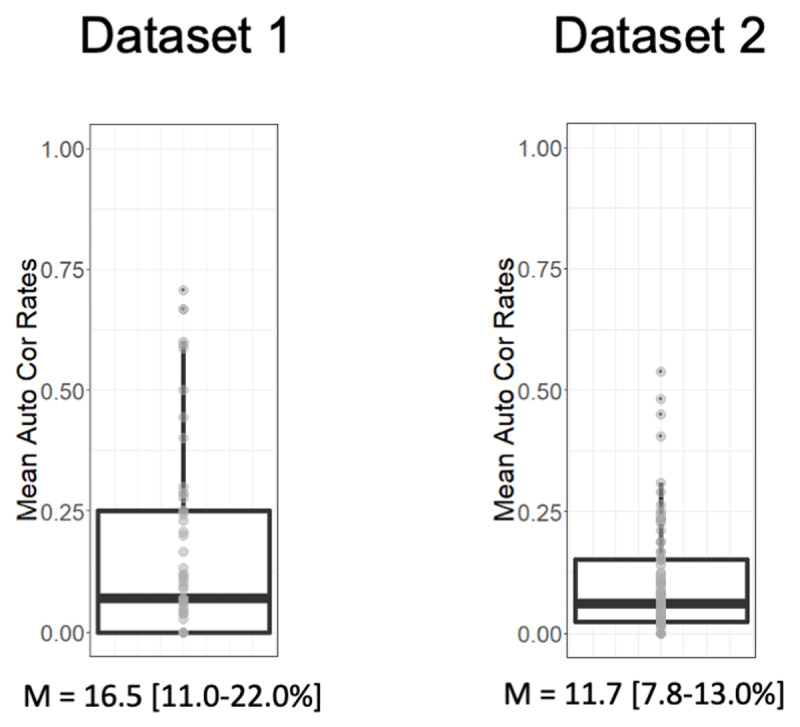
Distributions of the percentage of unreported correction attempts over subjects for each dataset. Median, Q1, and Q3 values are plotted as a boxplot, and given below each graph.

To summarize, there was clear evidence, across two datasets, for correction attempts that were not reported, suggesting the possibility that such correction attempts may have been carried out without conscious awareness. While the manipulations in dataset 2 exclude possibilities such as misunderstanding of instructions and mistakes in response selection, they cannot address a more theoretical alternative, namely that participants may have been conscious of a correction attempt but forgotten it by the time a report was due at the end of the trial. We evaluate this “memory failure” account using a positional analysis.

### Are unreported correction attempts memory failures?

Given the primacy and recency effects in memory (Ebbinghaus, 1913) and their well-established extension to the phonological buffer in production (Acheson & MacDonald, 2009; Page & Norris, 2009), a memory account of unreported repairs would predict the lowest probability of such repairs (i.e., the lowest rate of forgetting) at the beginning and end of the word, and a higher probability at the middle of the word. The observed data are plotted in ***Figure 4***. Position was coded as the distance between the repair and the final edge of the word, such that position 0 marks the last letter, position 1 the penultimate letter, and so on. A formal analysis with position as a predictor showed that the average position of the backspace in the word did not differ significantly between reported (*M* = 5.49) and unreported (*M* = 5.47) correction attempts, *Z* = 1.04, *p* = .30. Importantly, as can be seen in the figure, the pattern does not resemble the prediction of the memory account: the probability of not reporting a correction attempt was numerically highest in positions 7 and 0, i.e., at the beginning and end of the word, where such probability, if due to forgetting, should be the lowest. To attribute this to memory failure, one must assume *reversed* primacy and recency effects. These data rule out memory failure as a viable explanation for unreported correction attempts.

**Figure 4:**
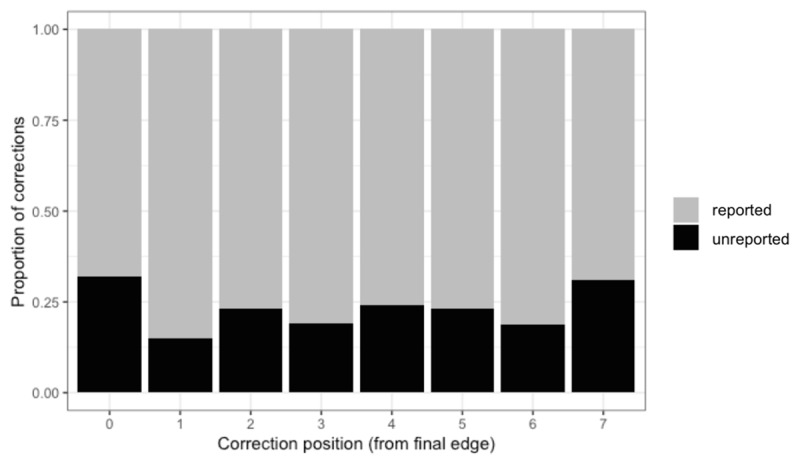
Stacked bars of reported (grey) and unreported (black) correction attempts by position. Position is coded as the distance from the final edge of the word, with position 0 being the final letter. Only responses that had the same number of characters as the target word were included, whether the final outcome was correct or not.

Before we move on to the properties of subconscious correction attempts, it is helpful to rule out another possibility, namely that participants may have had stored patterns of typing and correcting, as a chunk, for certain words that they automatically executed. This is reasonable for short and frequent words that are prone to consistent error patterns (e.g., “the” to “teh”). Our analysis showed that 46 words in these datasets were subject to only unreported correction attempts. By design, none of these words were shorter than 7 letters, none were function words, and their median log frequency was 2, meaning that they were not among the higher-frequency words in the lexicon. Moreover, the position of errors on these words was not consistent across participants, suggesting that the error patterns are not standards or highly predictable to be automated. The combination of these findings does not provide support for the idea of stored error + repair chunks that could be quickly and subconsciously executed as an integral pattern.

### Properties of unreported correction attempts

The analyses reported so far focused on establishing the existence of unreported correction attempts and the subconscious nature of such attempts. The next series of analyses focus on understanding the properties of these subconscious correction attempts.

*Stages of processing*. Because typing involves several levels of operation, typing errors may arise from different stages. Following previous works in typing (e.g., F. A. Logan, 1999; Rumelhart & Norman, 1982), G. D. Logan (2018) draws a distinction between finger selection errors (error keys adjacent to the target keys) and context-retrieval errors, i.e., those which happen prior to the launching of a finger movement command (often not adjacent to the target key). We compared 402 corrected adjacent errors to 772 corrected non-adjacent errors. The rate of unreported correction attempts was 23% vs. 18%, respectively, suggesting that unreported attempts were not restricted to errors arising from a certain level of processing. This difference between the two was also not significant, *Z* = 0.92, *p* = 0.36.

*Repair outcome*. Correction attempts can result in the correct response (successful repairs) or not. Of the 2023 correction attempts with correct outcomes, 23% were not reported. This was significantly higher than the 11% out of 1835 for attempts that did not result in the correct response, *Z* = –3.39, *p* < .001. This means that participants were more likely to fail to report an error and its attempt at correction if the final outcome had been correct.

*Timing of unreported correction attempts*. As the first-pass exploration of timing differences between reported and unreported correction attempts, we tested the differences between immediate and delayed repairs. Immediate repairs (i.e., those with a single backspace right after the error) were more common (2296) than the delayed repairs (i.e., with several backspaces to delete the letters typed after the error; 1558), and produced significantly more unreported correction attempts (19% vs. 13%, respectively; *Z* = –2.96, *p* = 0.003). Since this finding suggests that timing may be an important aspect of subconscious correction attempts, we followed up this analysis with more detailed analyses of the timing of reported vs. unreported correction attempts in immediate repairs.

The finding of longer RTs for error vs. correct words was replicated at the level of segments: error segments (234.94 ± 55 ms) had significantly longer IKIs than pre-error segments (171.78 ± 35 ms; *ß* = –62.0, *t* = –16.54, *p* < .001), suggesting the same general dynamics at the level of words and segments. The comparison of mean IKIs (i.e., IKIs averaged over all segments of the word) between reported (233.7 ± 85 ms) and unreported (206.2 ± 50 ms) correction attempts showed significantly longer times for the former (*ß* = 11.8, *t* = 5.72, *p* < .001). To further investigate the process from which this difference arose, we divided the timeline of a trial into four regions: 1) *Pre-error* was defined as all correct keystrokes before the error keystroke, 2) *Error* was defined as the error keystroke, 3) *Repair* was defined as the backspace keystroke and the following keystroke, which were analyzed separately. Finally, 4) *Post-repair* was defined as any keystroke following the repair region.

***Figure 5*** shows the duration of these regions in trials with reported and unreported repairs and ***Table 2*** reports the statistics. The results showed that the difference was confined to the Repair region. Both the backspace IKI and the new letter IKI were significantly faster for unreported than reported correction attempts (backspace IKI: *ß* = 18.0, *t* = 2.94, *p* = .0034; new letter IKI: *ß* = 11.7, *t* = 3.64, *p* < .001). Since repeated comparisons (i.e., different tests in the four zones) increase the probability of finding a significant effect by chance, it is appropriate to apply a correction for multiple comparisons to these results. Both effects remained significant after Bonferroni correction for multiple comparisons (corrected alpha-level = .008). None of the other regions showed significant differences between trials with and without reported correction attempts. This pattern of results shows that the effect is restricted to the repair process.

**Figure 5:**
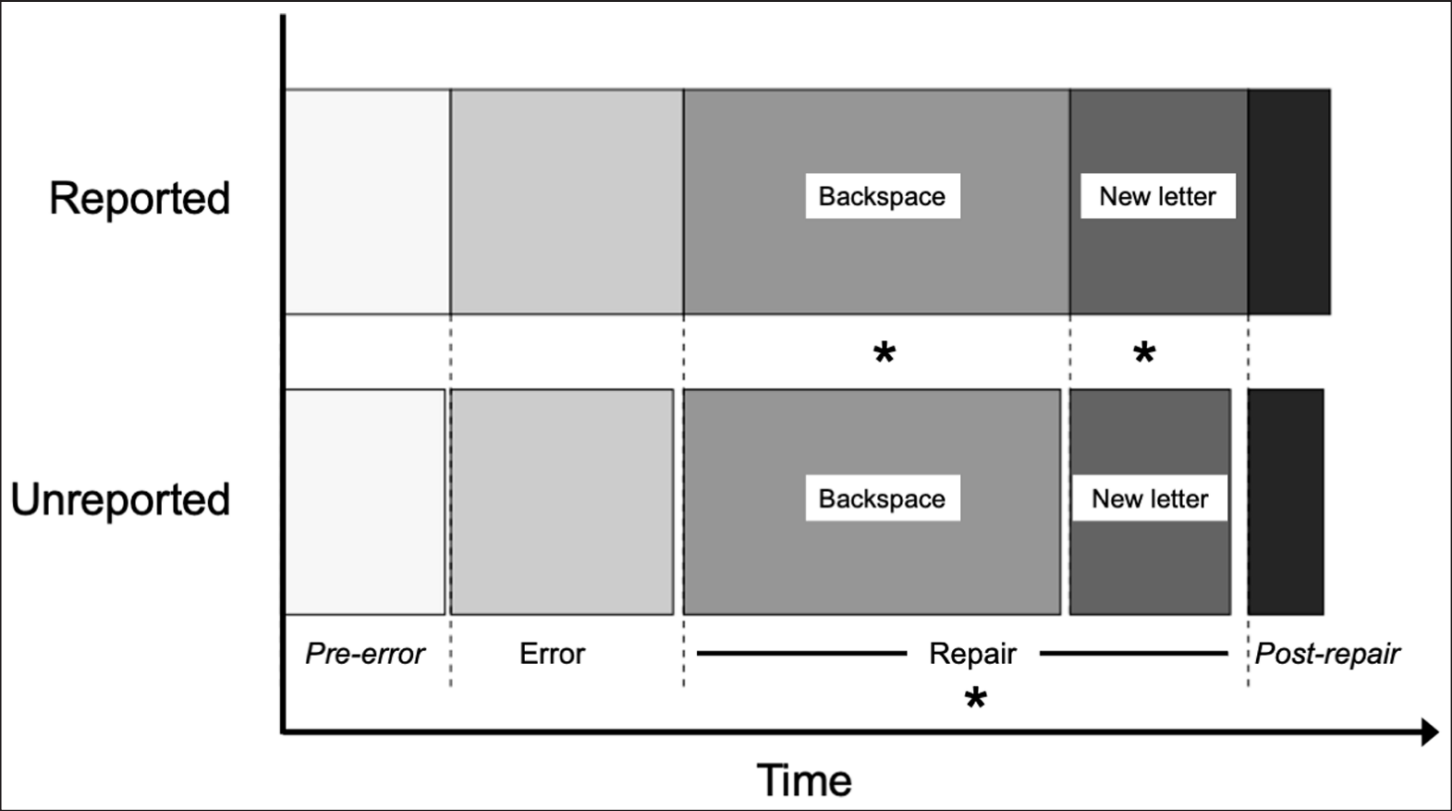
Timeline of trials with and without reported correction attempts. The IKI (or mean IKI if there are several keystrokes) is shown for each region. Asterisks indicates the region where the difference between reported and unreported correction attempts was significant.

**Table 2:** Results of statistical analyses comparing timing measures between reported and unreported correction attempts. All comparisons were run using mixed-effect linear models. Numbers in the parentheses are SD.


		REPORTED	NOT REPORTED	COHEN’S D	*ß*	*t*	*P*	*95%CI BOOTSTRAP*	

**General**	Mean IKI (ms)	233.7 (84.9)	206.2 (49.7)	0.409	11.8	5.72	<.001	7.39	15.9

**Pre-error**	Mean IKI before error (ms)	173.2 (39.0)	167.4 (52.8)	0.126	3.51	1.41	0.159	–1.61	8.59

**Error**	Error IKI (ms)	238 (63.5)	227.3 (116.8)	0.119	6.38	1.47	0.142	–1.94	14.6

**Repair**	Backspace IKI (ms)	394.8 (104.6)	385 (153.6)	0.076	18.0	2.94	0.0034	5.54	30.1

	IKI after backspace (ms)	182.3 (65.6)	163.8 (70.7)	0.271	11.7	3.64	<.001	5.2	18.5

**Post-correction**	Mean IKI after repair (ms)	83.7 (36.5)	76.7 (47.5)	0.167	2.71	0.971	0.332	–2.79	8.1


## Discussion

The purpose of this study was to determine that errors *can* be repaired subconsciously, and thus to test the sufficiency of accounts that rely on conscious initiation of repairs. Using a large corpus of typing errors, we reported clear evidence for correction (attempts) that were not reported, suggesting the possibility of repairing an error without conscious awareness. Specific manipulations in study 2 excluded possibilities such as misunderstanding of the instructions and mistakes in response selection. We further excluded the possibility that participants may have been conscious of a correction attempt but have forgotten it by the time they had to report it, by showing that the positional effects were incompatible with a memory failure account. These findings provide strong support for a mechanism for the existence of subconscious repairs. Next, we investigated three properties of conscious vs. subconscious repairs to help us define a repair mechanism. These three characteristics are summarized below, followed by a schematic model that accommodates them.

*Characteristic 1) Stage of processing*. Subconscious corrections were not confined to a certain stage of the system from which errors arose. Both adjacent and non-adjacent errors were subject to subconscious corrections at comparable rates. The existence of subconscious repairs in both error types, which roughly map onto errors of motor planning vs. segmental encoding in psycholinguistic models, suggests that subconscious repairs are not tied to a specific stage of processing. The repair model must thus accommodate a mechanism that is amenable to representations at different stages.

*Characteristic 2) Repair outcome*. Repairs may result in the correct response (successful repairs) or not. Our data show a stronger association between successful and subconscious repairs. Since neither of these variables have been experimentally manipulated, the direction of the effect is unclear. One interpretation is that the success of a repair makes it less likely to be reported. There is evidence that typists accept artificially corrected errors as their own correct responses (G. D. Logan & Crump, 2010; for similar findings in speech see Lind, Hall, Breidegard, Balkenius, & Johansson, 2015). Perhaps the final correct visual output helps erase any trace of having made an error (and subsequently a repair). This would be a plausible explanation if no visual information had been available until the outcome was displayed, as such situations have been shown to have a mild effect on error awareness and a more pronounced effect on correction attempts (Pinet & Nozari, 2020; Pinet & Nozari, 2021). But in this study, participants had access to visual information throughout the trial, so postulating a strong effect of the final visual outcome requires the assumption that all the online visual information has been erased. An alternative interpretation is that subconscious repairs are more likely to be successful, not because there is a causal relationship between the two, but rather because a third variable mediates both the probability of successful and subconscious repairs. We discuss this possibility further when proposing a model of repairs.

*Characteristic 3) Timing of subconscious corrections*. We found that the words containing a subconscious repair had significantly shorter durations than those containing a conscious repair. To pin down the source of this difference, we compared reported and unreported corrections within four regions: a) *pre-error*, b) *error*, c) *repair* (including the backspace and the new letter), and d) *post-repair*. The results were clear: in our dataset, the only significant differences in timing between conscious and subconscious repairs were observed in the repair zone itself. Both backspace and repair IKIs were significantly shorter in subconscious than conscious repairs. This finding excludes planning (pre-error), the error generation process (error), or post-error adjustments such as post-error slowing and conflict adaptation (post-error), as the locus of the difference between conscious and subconscious repairs.

One might object that although subconscious correction attempts were observed in this study, their rate was low. It is important to realize that the concept of statistical power, which is important in many comparisons, is not applicable here, because the point here is not to choose between one of the two alternatives, a conscious and a subconscious repair mechanism. It is clear that most people are conscious of most of their repairs most of the time. Rather, the question is whether a conscious repair mechanism is sufficient for explaining repairs in complex tasks. Any convincing demonstration of the occurrence of subconscious repairs rejects that position and invites an account that extends the repair process beyond mechanisms that must rely on conscious corrections.

Another objection could be that participants suffered lapses of attention, which prevented them from reporting their corrections. According to “when” such a lapse of attention may have taken place, the interpretations might vary. There are three possibilities: 1) a lapse of attention during repair is actually fully consistent with our account: repairs can be executed in the absence of conscious attention. 2) A lapse of attention after the repair but before the response, leaving them time to forget about it would look like the memory account, and we have rejected that possibility. 3) A lapse of attention during the response generation is not impossible, but the experimental setup, especially in Dataset 2, has made this very unlikely. The presentation of a correction prompt after no error was reported should appear as a strong unexpected event, and should really refocus participants on the task, assuming the information is consciously accessible to them. Nevertheless, 85% of participants in Dataset 2 still demonstrated evidence of subconscious corrections.

### Towards a model of repairs

The existence of subconscious repairs on a subset of trials is incompatible with models which assume that a repair must entail deliberate re-planning (e.g., G. D. Logan, 2018). Instead, the finding is better aligned with a process in which the detection of an error can trigger a local process of repair followed by the automatic resumption of production. The feasibility of subconscious processes for detection (Guenther, 2016; Hickok, 2012; Nozari et al., 2011) and resumption (G. D. Logan, 2018) has already been demonstrated. We propose to complete the picture with a repair mechanism that accommodates the three characteristics of the empirical data discussed above. ***Figure 6*** shows the schematic of the whole process.

**Figure 6:**
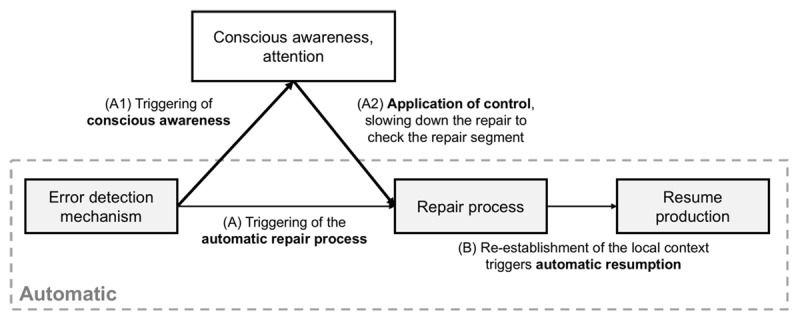
A schematic model of error detection, repair, and resumption. The process (A,B) can be completed without conscious awareness (dashed grey box). However, attentional processes are usually triggered by the detection mechanism (A1) and can override the automatic repair process or modify it (A2). (A) In typing, this corresponds to a backspace and a new letter. It replaces the current segment with the next most highly activated segment. (B) see G. D. Logan (2018) for an account of automatic resumption.

Errors arise when a target and one or more non-target segments are activated simultaneously. This triggers detection mechanisms (see Nozari, 2020, for a review). Detecting an error activates a repair routine. This routine may include a fixed command (e.g., press the backspace in typing), but its core process entails replacing the current representation with the next most highly activated representation. Since simultaneous activation of representations is found at different stages of processing from the message level to motor movements (e.g., Levelt, Roelofs, & Meyer, 1999; Servant, White, Montagnini, & Burle, 2015), this framework is compatible with characteristic 1. For the sake of simplicity, we use the case of segments as representations. The new segment then re-establishes the context for resuming production (G. D. Logan, 2018).

This pathway of detection, repair, resumption (the dashed grey box) can thus be completed without conscious awareness. But becoming aware of errors is desirable both for learning from one’s mistakes, and for potentially changing the path of subconscious repairs. To this end, upon detecting an error, a signal is sent to recruit attentional control (the part outside of the dashed grey box in ***Figure 6***). If recruited in time, attentional control overrides the subconscious repair process, i.e., it slows it down to check the error and the repair process, hence the longer IKIs in the repair region for reported vs. unreported repairs (characteristic 3).

When is attentional control bypassed? When the repair is over before attention is engaged. These are cases in which only one segment, other than the error, is highly activated, so the replacing segment is obvious (***Figure 7a***). In healthy systems, the competing segment is often the target segment, which leads to a successful repair. When more than one competing segment is activated at the time of repair, the system must first resolve the conflict between the different alternatives for replacement (***Figure 7b***). This process takes time (Nozari & Hepner, 2019), which buys time for attention to be engaged, and decreases the chance of subconscious repairs. The outcome of conflict resolution is also uncertain, i.e., the target has less chance of being selected, decreasing the chance of successful repairs. This dynamic provides a natural explanation for the link between subconscious and successful repairs (characteristic 2): subconscious and successful repairs are both a product of a certain state of the production system at the time of repair, without one directly causing the other.

**Figure 7:**
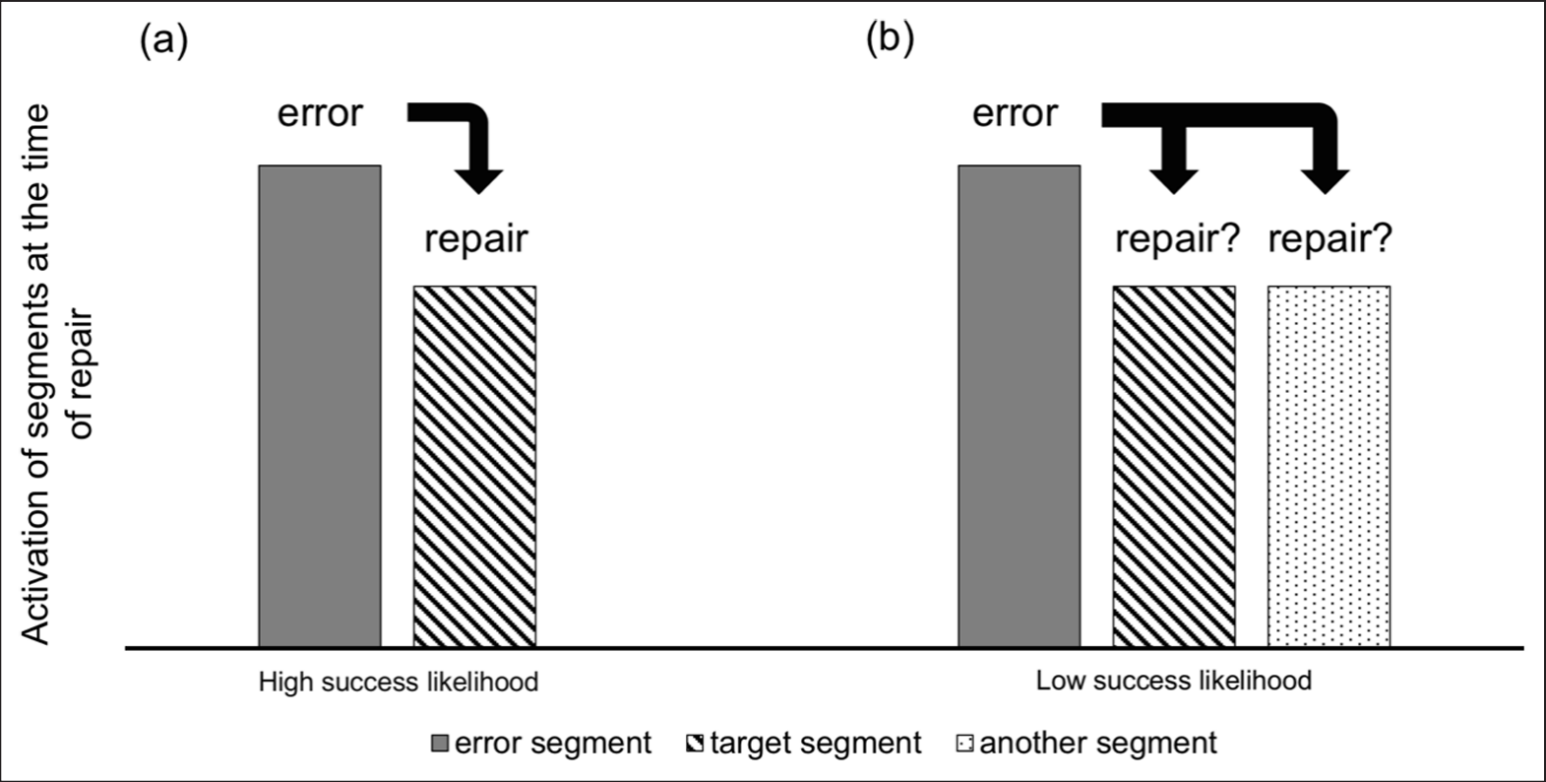
Schematics of activations of error and (potential) repair segments in trials with high (**a**) and low (**b**) rates of successful repairs. The repair process attempts to replace the error with the next most highly activated representation. In healthy systems, the target is almost always activated along with the error. If it is the only alternative, repair is quick and most likely successful (a). If more alternatives are simultaneously activated, the system must resolve the competition. This is time-consuming and the outcome is less certain to be the target (b).

## Conclusion

We provided empirical evidence for the presence of subconscious repairs in typing, and proposed a correction mechanism compatible with the characteristics of such repairs. This effort extends the accounts of automatic processing in complex tasks (e.g., Ficarella, Rochet, & Burle, 2019; G. D. Logan, 2018) to monitoring and repair processes in such tasks.
